# Non-linear relationship between the dietary inflammatory index and stroke risk in metabolically healthy obese individuals: an analysis of NHANES 1999-2023 data

**DOI:** 10.3389/fnut.2025.1608603

**Published:** 2025-07-04

**Authors:** Chuanwei Zhao, Yane Yang, Yunjie Yang, Wenzhou Yang, Lin Mu, Yan Jia

**Affiliations:** ^1^Department of Cardiology, The Second People’s Hospital of Baoshan, Baoshan, China; ^2^Kunming City Maternal and Child Health Hospital, Yunnan, China

**Keywords:** dietary inflammatory index, stroke, metabolically healthy obesity, NHANES, obesity

## Abstract

**Background:**

Studies on the relationship between the dietary inflammatory index (DII) and stroke risk in metabolically healthy obese (MHO) individuals are limited. This study aimed to explore the association between DII and stroke risk in MHO individuals, using data from the National Health and Nutrition Examination Survey (NHANES) 1999-2023.

**Methods:**

We performed a cross-sectional analysis of the NHANES, including 9872 MHO adults—defined as having a body mass index (BMI) ≥ 30 kg/m^2^ and no more than three metabolic abnormalities. Dietary intake was collected through 24-h recalls and weighted by the corresponding inflammatory effect coefficients, the sum of these weighted values yielded each participant’s DII score. Stroke status was ascertained from self-reported physician diagnosis recorded in the same survey cycle. Survey-weighted logistic regression and restricted cubic splines evaluated the DII–stroke association, while model performance was quantified with the area under the receiver operating characteristic (ROC) curve and decision-curve analysis (DCA).

**Results:**

A significant non-linear relationship was observed between DII and stroke risk. Below a DII score of 2.0, each 1-unit increase in DII was associated with a 32% higher stroke risk (OR: 1.32, 95% CI: 1.04–1.66; *p* = 0.02). Above this threshold, each 1-unit increase in DII was associated with a 38% reduction in stroke risk (OR: 0.62, 95% CI: 0.44–0.89; *p* = 0.01). The model’s predictive performance showed an AUC of 0.801 for the fully adjusted model.

**Conclusion:**

This study demonstrated a non-linear relationship between DII and stroke risk in MHO individuals, with a threshold effect at DII = 2.0. The DII may serve as a valuable predictor of stroke risk and guide dietary interventions in this population.

## 1 Introduction

Stroke is a leading cause of morbidity and mortality worldwide, and obesity is one of its most important modifiable risk factors (1). Large-scale cohort studies show a clear dose–response: Every 5 kg/m^2^ increase in body mass index (BMI) raises the incidence of both ischemic and hemorrhagic stroke by roughly 20–30% (2). This excess risk appears to be mediated, at least in part, by obesity-related systemic inflammation and endothelial dysfunction, even in the absence of overt metabolic syndrome (3).

Within the obese population, a distinction is often made between metabolically healthy obesity (MHO) and metabolically abnormal obesity (MAO) because their cardiometabolic outcomes differ (4). Operational definitions of MHO remain highly heterogeneous. For example, a large National Health and Nutrition Examination Survey (NHANES) analysis classified MHO as BMI ≥ 30 kg/m^2^ with no abnormal components of the Adult Treatment Panel III metabolic-syndrome criteria (5). An empirically derived definition from the UK Biobank proposed even stricter cut-offs—systolic blood pressure < 130 mmHg without antihypertensive therapy, waist-to-hip ratio < 0.95 in women or < 1.03 in men, and absence of type 2 diabetes—to identify the truly low-risk obese phenotype (6). A 2020 Endocrine Reviews synthesis cataloged more than 30 published criteria sets, showing that prevalence of MHO can range from 6 to 75% depending on which metabolic, inflammatory or insulin-sensitivity thresholds are applied, complicating direct comparisons between MAO and MHO groups (7). Although MHO individuals lack the classical metabolic derangements seen in MAO, they are not risk-free: Epidemiological data indicate that MHO still confers a higher probability of cardiovascular events—including stroke—than normal weight, pointing to additional, non-metabolic mechanisms (8, 9).

Diet plays a pivotal role in modulating systemic inflammation, a key driver of atherosclerosis and stroke (10). The Dietary Inflammatory Index (DII), a validated tool that quantifies the inflammatory potential of diets, has been associated with increased CVD risk across various populations (11). Pro-inflammatory diets, characterized by excessive intake of refined sugars, saturated fats, and processed meats, are linked to elevated inflammatory biomarkers, such as C-reactive protein (CRP) and interleukin-6 (IL-6), which in turn contribute to adverse cardiovascular outcomes (12). Conversely, anti-inflammatory diets, rich in fruits, vegetables, and omega-3 fatty acids, have been shown to mitigate these risks (13, 14). Despite extensive research on DII and CVD, critical gaps remain. First, prior studies have primarily focused on metabolically abnormal or general populations, neglecting the unique inflammatory dynamics in MHO individuals (15). Second, the relationship between DII and stroke risk in MHO individuals remains underexplored, particularly with respect to non-linear associations or threshold effects. Understanding these nuances is essential, as excessive inflammation might paradoxically trigger compensatory mechanisms or interact with metabolic resilience in MHO phenotypes (15), potentially influencing stroke risk in unexpected ways.

This study aims to investigate the relationship between DII and stroke risk in MHO individuals using data from the NHANES 1999-2023. By examining this relationship, we hope to provide insights into how diet, independent of metabolic abnormalities, influences stroke risk in MHO individuals and enhance the understanding of the inflammatory pathways involved in stroke pathogenesis.

## 2 Materials and methods

### 2.1 Study design

This study was designed as a retrospective cohort analysis using data from the NHANES, a comprehensive program that aims to evaluate the health and nutritional status of the U.S. civilian, non-institutionalized population. NHANES employs a sophisticated, multistage probability sampling method to ensure representative data collection. The survey gathers extensive demographic, socioeconomic, dietary, and health-related information through structured interviews, physical exams, and laboratory tests (16). The data collection process adheres to rigorous ethical standards, and the study was approved by the Research Ethics Review Board of the National Center for Health Statistics (NCHS). Informed written consent was obtained from all participants, ensuring both ethical and legal integrity in the study’s conduct.

### 2.2 Study population

The study population for this analysis was derived from the NHANES 1999–2023, a nationally representative survey designed to assess the health and nutritional status of the U.S. civilian, non-institutionalized population. The dataset included participants aged 20 years and older with a BMI ≥ 30 kg/m^2^. Individuals were classified as MHO or MAO based on the Harmonized Criteria for Metabolic Syndrome (MetS). MHO was defined as having ≤ 2 of the following criteria: Waist circumference (WC) ≥ 102 cm (men) or ≥ 88 cm (women), triglycerides (TG) ≥ 1.69 mmol/L, high-density lipoprotein cholesterol (HDL-C) < 1.04 mmol/L (men) or < 1.29 mmol/L (women), systolic blood pressure (SBP) ≥ 130 mmHg or diastolic blood pressure (DBP) ≥ 85 mmHg, and fasting plasma glucose (FPG) ≥ 5.6 mmol/L. Participants meeting ≥ 3 criteria were classified as MAO (17).

**FIGURE 1:**
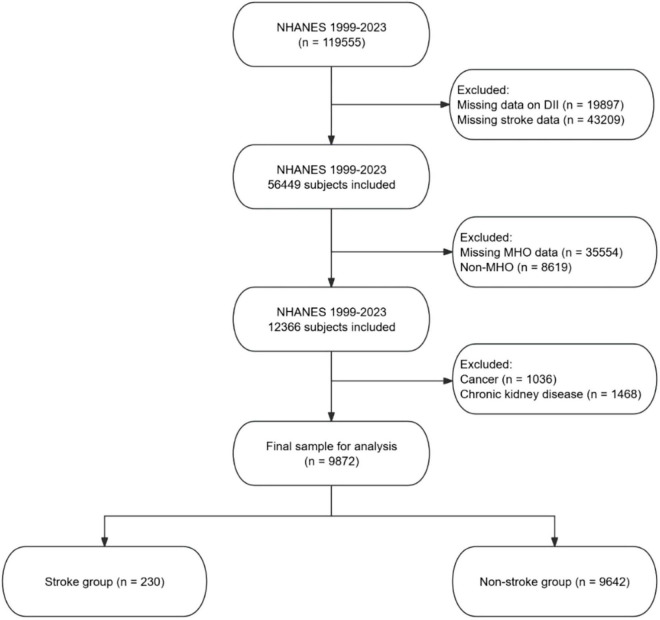
Flow chart of the study participants.

Participants were excluded if they had missing data on the DII or stroke data, had missing MHO data, or were classified as non-MHO. Additional exclusions were made for participants with cancer or chronic kidney disease (CKD), as these conditions could independently affect inflammation and stroke risk, potentially confounding the results. The final sample for analysis consisted of participants who were categorized into stroke and non-stroke groups. The screening process is detailed in Figure 1.

### 2.3 Assessment of dietary information

The DII is a tool designed to assess the inflammatory potential of dietary intake. It incorporates 45 food parameters, each assigned a specific DII score based on its influence on six key inflammatory biomarkers: IL-1β, IL-4, IL-6, IL-10, TNF-α, and CRP (18). In the NHANES study, DII calculation primarily relies on 24-h dietary recall, where participants report the types, amounts, and timing of all foods and beverages consumed in the previous day (19). These intake data are then compared to global food composition databases to standardize the intake of each food or nutrient. The resulting standardized data are transformed into percentile scores to mitigate the effects of skewed dietary data. Each food component’s percentile score is weighted by its inflammatory effect score, indicating its proinflammatory or anti-inflammatory potential. The sum of these weighted scores provides the individual’s overall DII score (18).

The DII has been validated in various studies across different populations, such as postmenopausal women (20), African Americans (21), and individuals with chronic conditions (22), confirming its reliability and applicability in evaluating diet-related inflammation in diverse groups. In this study, DII computation includes the intake of 28 specific nutrients, including carbohydrates, protein, total fat, alcohol, fiber, monounsaturated fatty acids (MUFA), polyunsaturated fatty acids (PUFA), vitamins A, B1, B2, B6, B12, C, D, E, niacin, riboflavin, folic acid, magnesium, iron, zinc, selenium, beta-carotene, caffeine, and energy. Notably, the DII remains valid even when fewer than 30 nutrients are included (23). In the scoring system, anti-inflammatory foods and nutrients are assigned negative scores, while proinflammatory ones receive positive scores (24). It is important to note that the impact of some food components may vary depending on the quantity consumed. For example, small amounts of alcohol may exert anti-inflammatory effects, resulting in a negative score, while larger amounts can have proinflammatory effects, leading to a positive score (25). This dose-dependent effect is a critical feature of the DII calculation, capturing how varying quantities of the same food component influence its inflammatory potential.

### 2.4 Diagnosis of stroke

In this study, stroke diagnosis was based on participants’ self-reported responses to a specific question in the NHANES survey. During data collection, participants were asked, “Has a physician or other health professional ever told you that you had a stroke?” Based on their affirmative or negative responses, participants were categorized into the “Stroke” group or the “Non-stroke” group.

### 2.5 Covariates

In this study, participants were categorized into the following racial/ethnic groups: Mexican American, non-Hispanic Black, non-Hispanic White, other Hispanic, and other races. Educational attainment was classified into two categories: Below high school and high school or above. Marital status was divided into three groups: Married or living with partner, never married, and widowed/divorced/separated. The poverty-income ratio (PIR) was used to adjust income levels for economic inflation and household size. Smoking habits, alcohol consumption status, physical activity, and medical histories (including diabetes, hypertension, and use of antihypertensive agents, glucose-lowering drugs, lipid-lowering agents, and antiplatelet medications) were collected via self-administered questionnaires. Smoking status was categorized as never smoked, former smoker, or current smoker. Alcohol consumption was classified as active drinker or non-active drinker. Physical activity levels were assessed based on participation in various activities: Walking/cycling, housework/yard work, muscle-strengthening exercises, work-related activities, and recreational activities. A detailed questionnaire recorded the frequency and duration of these activities. Physical activity data were expressed as weekly Metabolic Equivalent of Task (MET) values, calculated by multiplying the MET value of each activity by its weekly duration. WC, body weight, and height were measured according to standardized protocols from the Anthropometry Standardization Reference Manual (26). Blood pressure measurements followed the latest American Heart Association guidelines (27). Laboratory evaluations included: Estimated glomerular filtration rate (eGFR), FPG, hemoglobin A1c (HbA1c), serum creatinine (SCR), TG, TC, HDL-C, and low-density lipoprotein cholesterol (LDL-C). CKD status was determined using the 2024 CKD Epidemiology Collaboration (CKD-EPI) creatinine equation. CKD diagnosis was defined as an eGFR < 60 mL/min/1.73 m^2^ or urine albumin-to-creatinine ratio (UACR) > 30 mg/g (28).

### 2.6 Statistical analysis

All analyses adhered to NHANES protocols with sampling weights applied according to survey cycles:“WTDR4YR” for 1999-2002 and “WTDRD1” for 2003-2023. Participants were stratified into stroke and non-stroke groups based on physician-diagnosed stroke history obtained through self-report. Continuous variables were expressed as mean ± standard deviation (SD) and analyzed using Studenttiot-test for normally distributed variables (Shapiro-Wilk *p* ≥ 0.05) or Wilcoxon rank-sum test for non-normally distributed data. Categorical variables were reported as frequencies with percentages, compared by χ^2^ test or Fisher’s exact test when expected cell counts were below 5.

Prior to model construction, variables with variance inflation factors (VIF) > 5 were excluded to mitigate multicollinearity. Multivariable logistic regression models were employed to examine the association between the DII and stroke risk through three progressively adjusted models: Model 1 (unadjusted); Model 2 adjusted for demographic factors (age, sex, race); Model 3 additionally adjusted for education level, marital status, PIR, total physical activity, smoking status, and alcohol consumption. DII was analyzed both as a continuous variable and in quartiles, with the first quartile serving as the reference. Restricted cubic splines (RCS) with three prespecified knots at the 5th, 50th, and 95th DII percentiles evaluated non-linear relationships, confirmed through likelihood ratio testing. Upon identifying a significant non-linear term (*p* < 0.05), maximum likelihood estimation determined threshold effects, followed by piecewise regression analysis. Subgroup stratification by sex, age groups (< 60 vs. ≥ 60 years), race, smoking status, and alcohol consumption included interaction term assessment. Model discrimination was quantified through receiver operating characteristic (ROC) curves analyzed by DeLongcri*Z*-test and clinical utility evaluated via decision curve analysis (DCA). Less than 5% missing data were imputed using predictive mean matching (29). All computations were performed in R version 4.4.2 with statistical significance defined as two-tailed *p* < 0.05.

## 3 Results

### 3.1 Baseline characteristics of participants

Among 9,872 MHO individuals, significant disparities were observed between stroke (*n* = 230) and non-stroke groups (*n* = 9,642) (Table 1). The stroke group exhibited older age, higher proportions of non-Hispanic Black and Mexican American individuals, lower educational attainment, increased rates of widowhood/divorce, and reduced PIR. Metabolic profiles showed elevated prevalence of hypertension and diabetes, higher fasting glucose and HbA1c levels, and significantly greater DII scores. Medication use for hypertension, hypoglycemia, dyslipidemia, and antiplatelet therapy was markedly higher in the stroke group. No differences were detected in physical activity, smoking status, SCR, TG, TC, HDL-C, LDL-C, BMI, or WC.

**TABLE 1:** Baseline characteristics stratified by stroke status.

Characteristics	Total (*N* = 9,872)	Stroke group (*n* = 230)	Non-stroke group (*n* = 9,642)	*P*-value
Age, years	42.51 ± 0.22	42.28 ± 0.22	54.75 ± 1.64	< 0.001
Gender, n (%)				0.18
Female	5,717 (53.43)	5,577 (53.30)	140 (60.02)	
Male	4,155 (46.57)	4,065 (46.70)	90 (39.98)	
Race, n (%)				0.01
Mexican American	1,868 (10.89)	1,840 (11.00)	28 (5.37)	
Non–Hispanic Black	2,805 (16.84)	2,721 (16.69)	84 (25.25)	
Non–Hispanic White	3,736 (59.80)	3,657 (59.88)	79 (55.57)	
Other Race	1,463 (12.46)	1,424 (12.43)	39 (13.80)	
Education level, n (%)				< 0.001
Below high school	2,225 (15.25)	2,156 (15.20)	69 (18.00)	
High school graduate or GED	2,365 (25.41)	2,290 (25.06)	75 (43.63)	
Some college or above	5,282 (59.34)	5,196 (59.74)	86 (38.37)	
Marital status, n (%)				< 0.001
Married or Living with a partner	6,003 (62.12)	5,868 (62.21)	135 (57.26)	
Never married	2,067 (21.97)	2,047 (22.22)	20 (8.75)	
Widowed, divorced, or separated	1,802 (15.91)	1,727 (15.57)	75 (33.99)	
PIR	2.90 ± 0.04	2.91 ± 0.04	2.41 ± 0.18	0.01
Physical activity total METs/week	4,422 ± 110	4,421 ± 110	4,456 ± 636	0.96
Smoke				0.26
Former	2,155 (22.04)	2,082 (22.01)	73 (23.33)	
Never	5,960 (59.18)	5,854 (59.30)	106 (52.49)	
Now	1,757 (18.78)	1,706 (18.68)	51 (24.18)	
Alcohol use, n (%)				0.003
Active alcohol user	7,235 (77.46)	7,098 (77.69)	137 (65.31)	
Non-active alcohol user	2,637 (22.54)	2,544 (22.31)	93 (34.69)	
Hypertension, n (%)				< 0.001
No	6,603 (69.84)	6,530 (70.35)	73 (42.78)	
Yes	3,269 (30.16)	3,112 (29.65)	157 (57.22)	
DM, n (%)				< 0.001
No	8,636 (90.10)	8,479 (90.49)	157 (69.43)	
Yes	1,236 (9.90)	1,163 (9.51)	73 (30.57)	
eGFR, mL/min/1.73 m^2^	101.03 ± 0.36	101.19 ± 0.36	92.64 ± 2.02	< 0.001
FPG, mg/dL	100.14 ± 0.31	99.97 ± 0.31	109.02 ± 2.92	0.003
HbA1c, %	5.56 ± 0.01	5.55 ± 0.01	5.84 ± 0.09	0.001
SCR, mg/dL	0.83 ± 0.00	0.83 ± 0.00	0.83 ± 0.01	0.68
TG, mg/dL	165.01 ± 1.64	165.25 ± 1.68	152.31 ± 9.21	0.17
TC, mg/dL	197.69 ± 0.62	197.75 ± 0.63	194.41 ± 3.13	0.3
HDL-C, mg/dL	50.78 ± 0.21	50.74 ± 0.21	52.79 ± 1.17	0.08
LDL-C, mg/dL	113.79 ± 0.46	113.84 ± 0.47	111.23 ± 3.39	0.45
BMI, kg/m^2^	35.49 ± 0.08	35.49 ± 0.08	35.69 ± 0.46	0.67
WC, cm	112.38 ± 0.18	112.35 ± 0.18	113.71 ± 1.35	0.32
DII	1.63 ± 0.03	1.62 ± 0.03	2.08 ± 0.11	< 0.001
Antihypertensive drugs, n (%)				< 0.001
No	7,708 (80.86)	7,624 (81.49)	84 (47.28)	
Yes	2,164 (19.14)	2,018 (18.51)	146 (52.72)	
Hypoglycemic agents, n (%)				< 0.001
No	9,100 (93.85)	8,929 (94.17)	171 (76.48)	
Yes	772 (6.15)	713 (5.83)	59 (23.52)	
Lipid lowering drugs, n (%)				< 0.001
No	8,631 (88.88)	8,492 (89.38)	139 (62.16)	
Yes	1,241 (11.12)	1,150 (10.62)	91 (37.84)	
Antiplatelet drugs, n (%)				< 0.001
No	9,703 (98.70)	9,515 (98.98)	188 (83.96)	
Yes	169 (1.30)	127 (1.02)	42 (16.04)	

Continuous measures are presented as mean ± standard deviation (SD), with categorical data summarized using absolute frequencies and relative percentages. Group comparisons were conducted using Student’s *t*-test or Wilcoxon rank-sum test for continuous variables, and chi-square test or Fisher’s exact test for categorical variables, as appropriate. A two-sided *P*-value < 0.05 was considered statistically significant. GED, general educational development; PIR, poverty–income ratio; MET, metabolic equivalent of task; BMI, body mass index; WC, waist circumference; DM, diabetes mellitus; eGFR, estimated glomerular filtration rate; FPG, fasting plasma glucose; HbA1c, hemoglobin A1c; SCR, serum creatinine; TG, triglycerides; TC, total cholesterol; HDL-C, high-density lipoprotein cholesterol; LDL-C, low-density lipoprotein cholesterol; DII, dietary inflammatory index.

### 3.2 Association between DII and stroke risk in MHO participants

Table 2 presents the weighted multivariable logistic regression analysis of the DII and stroke risk among MHO participants. In the unadjusted model (Model 1), each 1-unit increase in DII was associated with a 17% higher stroke risk (OR = 1.17, 95%CI:1.08–1.27, *p* < 0.001). Adjusting for demographics (Model 2) slightly attenuated the association (OR = 1.16, 95%CI:1.07–1.25, *p* < 0.001), which remained significant after further adjustment for sociobehavioral factors (Model 3: OR = 1.12, 95%CI:1.03–1.21, *p* = 0.01). Using the first quartile (Q1) as reference, the third quartile (Q3) demonstrated consistently elevated risks across all models (Model 3: OR = 2.71, 95%CI:1.49–4.91, *p* = 0.001), whereas the highest quartile (Q4) showed non-significant association in the fully adjusted model (OR = 1.65, 95%CI:0.97–2.82, *p* = 0.07). Dose-response relationships were confirmed by trend tests (P for trend: Model 1 = 0.002, Model 3 = 0.037).

**TABLE 2:** Weighted logistic regression model of stroke risk associated with DII in MHO participants.

Characteristic	Model 1		Model 2		Model 3	
	OR (95% CI)	*p*	OR (95% CI)	*p*	OR (95% CI)	*p*
DII (Continuous)	1.17 (1.08, 1.27)	< 0.001	1.16 (1.07, 1.25)	< 0.001	1.12 (1.03, 1.21)	0.01
Q1	Reference	–	Reference	–	Reference	–
Q2	1.79 (0.99, 3.26)	0.06	1.87 (1.04, 3.38)	0.04	1.68 (0.94, 3.01)	0.08
Q3	2.88 (1.63, 5.09)	< 0.001	3.08 (1.72, 5.49)	< 0.001	2.71 (1.49, 4.91)	0.001
Q4	2.05 (1.20, 3.50)	0.01	1.93 (1.12, 3.32)	0.02	1.65 (0.97, 2.82)	0.07
P for trend		0.002		0.005		0.037

Model 1: Unadjusted. Model 2: Adjusted for age, sex, and race. Model 3: Adjusted for age, sex, race, education level, marital status, PIR, total physical activity (MET), smoking status, and alcohol use. *P*-values were derived from survey-weighted logistic regression models. A two-sided *P*-value < 0.05 was considered statistically significant. DII, dietary inflammatory index; MHO, metabolically healthy obese; OR, odds ratio; CI, confidence interval; PIR, poverty-income ratio.

### 3.3 Restricted cubic spline and threshold effect analysis

**FIGURE 2:**
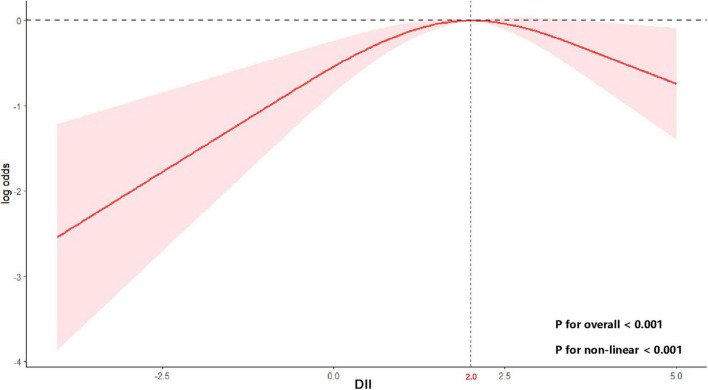
Non-linear relationship between DII and stroke risk in MHO participants. Curve and 95% confidence bands derived from a restricted cubic spline model using survey-weighted logistic regression (Model 3). A two-sided *P*-value < 0.05 was considered statistically significant.

Using RCS with multivariable-adjusted logistic regression models, we examined the dose-response relationship between the DII and stroke risk in MHO participants. A significant non-linear association was observed across the DII spectrum (P for non-linearity < 0.001) (Figure 2). Adjusted spline curves demonstrated an initial increase in stroke risk with rising DII levels, followed by an unexpected risk reduction beyond a threshold value. Threshold analysis identified an inflection point at DII = 2.0 (log-likelihood ratio test, *p* < 0.001). Below this threshold, each 1-unit increase in DII was associated with a 32% higher stroke risk (OR: 1.32, 95% CI: 1.04–1.66; *p* = 0.02). Conversely, above the threshold, each 1-unit increase in DII corresponded to a 38% risk reduction (OR: 0.62, 95% CI: 0.44–0.89; *p* = 0.01) (Table 3).

**TABLE 3:** Threshold effect analysis of DII on stroke risk in MHO participants.

	OR (95% CI)	*P-*value
DII < 2	1.32 (1.04, 1.66)	0.02
DII ≥ 2	0.62 (0.44, 0.89)	0.01
P for Log-likelihood ratio		< 0.001

Adjusted for age, sex, race, education level, marital status, PIR, total physical activity (MET), smoking status, and alcohol use. DII, dietary inflammatory index; MHO, metabolically healthy obese; OR, odds ratio; CI, confidence interval. The logistic regression model was used to estimate OR and 95% CI. A two-sided *P-*value < 0.05 was considered statistically significant.

### 3.4 Subgroup analysis

Table 4 demonstrates heterogeneous associations between the DII and stroke risk across subgroups of MHO participants. Among individuals aged < 60 years, DII < 2 was associated with a 41.8% increased stroke risk (OR = 1.418, 95% CI: 1.039 stroke *p* = 0.028), while no significant association was observed for DII ≥ 2. Males exhibited a 58.1% reduced risk at DII ≥ 2 (OR = 0.419, 95% CI: 0.203– 0.862, *p* = 0.019), though the gender interaction was non-significant (P for interaction = 0.165). Non-Hispanic White individuals showed elevated risk at DII < 2 (OR = 1.389, 95% CI: 1.013 – 1.904, *p* = 0.042), whereas other racial/ethnic groups displayed no significant trends. Current smokers had a 62.6% higher risk at DII < 2 (OR = 1.626, 95% CI: 1.026 – 2.578, *p* = 0.039), and non-active alcohol users demonstrated a 50.6% risk reduction at DII ≥ 2 (OR = 0.494, 95% CI: 0.274 – 0.888, *p* = 0.019). Despite subgroup-specific associations, all interaction tests (P for interaction > 0.05) were non-significant, indicating no statistically discernible heterogeneity in the DII-stroke risk relationship across subgroups.

**TABLE 4:** Stratified analysis of DII and stroke risk in MHO participants by DII categories.

Characteristic	DII (overall)		DII < 2		DII ≥ 2	
	OR (95% CI)	*p*	OR (95% CI)	*p*	OR (95% CI)	*p*
**Age group**	**P for interaction = 0.430**		**P for interaction = 0.436**		**P for interaction = 0.454**	
< 60 y	1.078 (0.963, 1.207)	0.192	1.418 (1.039, 1.935)	0.028	0.553 (0.301,1.016)	0.056
≥ 60 y	1.152 (1.004, 1.323)	0.045	1.139 (0.754, 1.722)	0.533	0.706 (0.425, 1.171)	0.175
**Sex**	**P for interaction = 0.767**		**P for interaction = 0.212**		**P for interaction = 0.165**	
Female	1.090 (0.969, 1.225)	0.150	1.572 (1.067, 2.315)	0.022	0.734 (0.483, 1.114)	0.145
Male	1.132 (0.981, 1.307)	0.088	1.140 (0.832, 1.562)	0.412	0.419 (0.203, 0.862)	0.019
**Race**	**P for interaction = 0.283**		**P for interaction = 0.331**		**P for interaction = 0.744**	
Mexican American	1.434 (1.009, 2.038)	0.045	1.594 (0.648, 3.918)	0.305	1.093 (0.399, 2.995)	0.860
Non–Hispanic Black	0.999 (0.864, 1.155)	0.984	0.987 (0.731, 1.333)	0.932	0.754 (0.466, 1.222)	0.250
Non–Hispanic White	1.126 (0.984, 1.290)	0.085	1.389 (1.013, 1.904)	0.042	0.575 (0.326, 1.013)	0.055
Other race	1.143 (0.935, 1.397)	0.189	1.757 (0.731, 4.220)	0.205	0.486 (0.157, 1.509)	0.209
**Smoke**	**P for interaction = 0.822**		**P for interaction = 0.896**		**P for interaction = 0.984**	
Former	1.086 (0.928, 1.271)	0.300	1.272 (0.809, 2.000)	0.296	0.519 (0.272, 0.991)	0.047
Never	1.151 (0.999, 1.326)	0.052	1.291 (0.920, 1.813)	0.138	0.669 (0.403, 1.111)	0.120
Now	1.043 (0.870, 1.249)	0.650	1.626 (1.026, 2.578)	0.039	0.600 (0.311, 1.158)	0.126
**Alcohol use**	**P for interaction = 0.380**		**P for interaction = 0.999**		**P for interaction = 0.287**	
Active alcohol user	1.113 (0.998, 1.241)	0.055	1.322 (1.006, 1.736)	0.045	0.702 (0.455, 1.085)	0.110
Non-active alcohol user	1.082 (0.940, 1.246)	0.271	1.285 (0.845, 1.953)	0.239	0.494 (0.274, 0.888)	0.019

The logistic regression was used to estimate the OR and 95% CI, with adjustments for confounding factors. A two-sided *P*-value < 0.05 was considered statistically significant. DII, dietary inflammatory index; MHO, metabolically healthy obese; OR, odds ratio; CI, confidence interval.

### 3.5 Predictive performance evaluation

**FIGURE 3:**
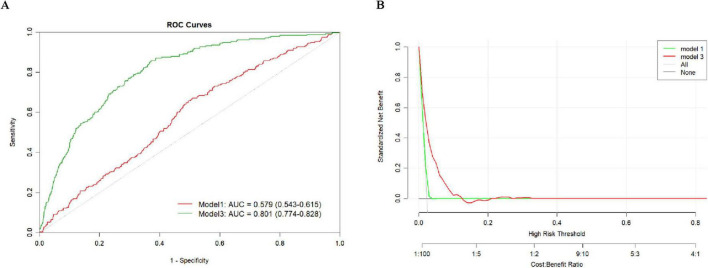
Predictive performance of DII for stroke risk in MHO participants. **(A)** ROC and AUC, **(B)** Decision curve analysis.

Figure 3 presents the predictive performance of the DII for stroke risk in MHO participants. Figure 3A shows the ROC curves for Model 1 and Model 3 in predicting stroke risk, along with their corresponding AUC values. The AUC for Model 1 was 0.579 (95% CI: 0.543–0.615), while Model 3 showed an AUC of 0.801 (95% CI: 0.774–0.828). Figure 3B illustrates the DCA, which was used to assess the clinical value of Model 1 and Model 3. DCA helps to understand the net benefit of using these models for stroke prediction at different risk thresholds. The green and red curves represent the net benefits of Model 1 and Model 3, respectively, showing their trends across varying risk thresholds. The net benefit of both models decreases as the risk threshold increases. Overall, Model 3 outperforms Model 1 in predicting stroke risk, particularly at higher risk thresholds.

## 4 Discussion

### 4.1 Main findings

This study utilized data from the 1999-2023 NHANES to explore the non-linear relationship between the DII and stroke risk in MHO individuals. A significant threshold effect was identified at a DII score of 2.0. Below this threshold, each 1-unit increase in DII was associated with a 32% higher stroke risk, whereas above this threshold, each 1-unit increase in DII corresponded to a 38% reduction in stroke risk. Subgroup analyses demonstrated significant heterogeneity in the DII-stroke relationship across various age, sex, race, and smoking status groups. Predictive performance evaluation showed that the DII exhibited strong discriminatory power in stroke risk prediction, with an AUC of 0.801 in the fully adjusted model. These findings suggest that DII may serve as a valuable tool for predicting stroke risk in MHO individuals and hold potential clinical implications for personalized dietary recommendations.

### 4.2 Previous research on DII and CVD

Prior studies have highlighted the significant relationship between the DII and CVD, particularly stroke. A systematic review and meta-analysis indicated that higher DII scores are associated with an increased risk of CVD, emphasizing the impact of dietary inflammation on overall cardiovascular health (30). In the US general population, a non-linear and positive association between DII and stroke has been identified, supporting the notion that higher DII levels correlate with greater stroke risk (31). However, in women, the association between DII and cardiovascular disease risk was not significant when supplements were considered, indicating that other factors may moderate this relationship (11). Further research found that DII was positively correlated with all-cause mortality in patients with atherosclerotic cardiovascular disease, suggesting that a pro-inflammatory diet could increase mortality in these patients (32). In individuals with type 2 diabetes, DII’s role in predicting cardiovascular disease risk is highlighted, showcasing the potential for precision medicine in understanding the role of dietary inflammation (33). Studies conducted in the US and European populations have also demonstrated the significant association between DII and cardiovascular disease risk, underlining the importance of dietary patterns in modulating inflammation and cardiovascular health (34). Additionally, in Asian populations, DII has been linked to stroke risk, contributing to the understanding of dietary inflammation across different ethnic groups (35). Older adults, who are particularly vulnerable to stroke, were found to have an increased cardiovascular risk due to higher DII, emphasizing the need for tailored dietary strategies in aging populations (36). In obese individuals, another high-risk group for stroke, dietary inflammation was found to significantly influence cardiovascular disease risk, further reinforcing the need for managing dietary factors in obesity (37). The combined effect of smoking and dietary inflammation has been explored, highlighting how lifestyle factors like smoking can exacerbate the negative impact of dietary inflammation on cardiovascular health (38). Finally, in individuals with hypertension, a major stroke risk factor, DII was found to influence cardiovascular disease risk, suggesting that managing inflammation through diet could be a key intervention strategy in hypertensive patients (10). These findings collectively underscore the role of dietary inflammation in cardiovascular disease risk, particularly stroke, across different populations.

### 4.3 Explanation of results

The study’s results reveal a non-linear relationship between the DII and stroke risk. In certain specific subgroups—such as males, non-alcohol users, and former smokers—higher DII values appear to have a protective effect. This seems counterintuitive because pro - inflammatory diets are usually associated with an increased risk of stroke. This can be attributed to a variety of factors. First, MHO individuals may require a certain level of inflammation to maintain vascular health, as diets with lower DII might not provide sufficient pro-inflammatory stimuli needed for vascular resilience, leading to an increased stroke risk. In contrast, higher DII values could activate compensatory anti-inflammatory mechanisms, mitigating the risk of stroke (39). Second, the threshold effect suggests that moderate inflammation, which is indicated by higher DII, might be beneficial in MHO individuals, as it activates protective mechanisms in the body (13). Third, the lack of metabolic dysfunction in MHO individuals means the effects of dietary inflammation on stroke risk may be more pronounced compared to metabolically abnormal individuals, with the inflammatory potential of the diet playing a key role in maintaining cardiovascular health (9). Additionally, the balance between pro-inflammatory and anti-inflammatory foods in the diet may contribute to this paradoxical effect, where high DII scores reflect a diet that includes beneficial nutrients, such as omega-3 fatty acids and antioxidants, which counterbalance the harmful effects of inflammation (40, 41). Furthermore, lifestyle factors like physical activity, which has anti-inflammatory effects, may help reduce stroke risk in MHO individuals despite a higher DII (42). Finally, genetic predispositions may also play a role in how MHO individuals respond to dietary inflammation, with certain genetic factors potentially protecting against the harmful effects of inflammation, thereby reducing stroke risk despite higher DII values (43).

### 4.4 Mechanisms linking DII to stroke risk

The relationship between DII and stroke risk is underpinned by several mechanisms involving diet-induced inflammation and its effects on vascular health. A high-DII diet, characterized by pro-inflammatory foods such as processed meats, refined sugars, and trans fats, promotes chronic low-grade inflammation, which plays a crucial role in endothelial dysfunction (44, 45). This dysfunction impairs the ability of blood vessels to regulate tone and flow, leading to an increased risk of thrombus formation and, ultimately, stroke (46). Additionally, high-DII diets contribute to oxidative stress, a process where reactive oxygen species damage cells and accelerate atherosclerosis by promoting the oxidation of LDL-C, This process leads to plaque buildup in arteries, which can rupture, causing blood clots that obstruct cerebral blood flow, resulting in ischemic stroke (47). The pro-inflammatory nature of a high-DII diet also disrupts lipid metabolism, often resulting in dyslipidemia with elevated total cholesterol and triglycerides and reduced HDL-C, These lipid abnormalities further exacerbate atherosclerosis, making stroke more likely (48). Moreover, high-DII diets increase the production of pro-inflammatory cytokines, such as interleukin-6 and tumor necrosis factor-alpha, which trigger vascular inflammation and promote hypercoagulability (49). This increases the likelihood of clot formation, contributing to thrombotic strokes. Immune system activation is another key mechanism, as prolonged inflammation leads to elevated levels of inflammatory markers, such as CRP, which promote vascular inflammation and compromise blood-brain barrier integrity, increasing the risk of both ischemic and hemorrhagic strokes (50, 51). Additionally, diet-induced inflammation leads to endothelial dysfunction and elevated blood pressure, a significant risk factor for stroke (52). Besides diet-induced inflammation, Inadequate physical activity — defined as performing less than 150 min per week of moderate-to-vigorous physical activity (MVPA) — independently worsens endothelial function and amplifies obesity-related inflammation. A large meta-analysis of prospective studies reported that people in the lowest physical-activity category had about a 20% higher risk of total, ischemic, and hemorrhagic stroke compared with their more active peers, and each incremental step of roughly 500 MET-min per week was associated with a further 10% change in risk (53). Regular MVPA also lowers circulating C-reactive protein and interleukin-6, key mediators along the diet–inflammation pathway (54). Current World Health Organization guidelines recommend accumulating 150–300 min of MVPA weekly (about 600 MET-min) to obtain cardiovascular and cerebrovascular benefits (55). Together, these data imply that the stroke risk associated with a pro-inflammatory diet (high DII) may be magnified when physical-activity volume falls below this threshold, underscoring the need to address both diet and physical activity in prevention strategies. Importantly, emerging evidence from genome-wide association studies (GWAS) suggests that genetic variants related to adiposity and inflammatory signaling can modify an individual’s inflammatory response to dietary exposures. Carriers of pro-inflammatory alleles exhibit higher CRP and IL-6 levels when consuming high-DII diets and display greater susceptibility to stroke, implying a gene–diet interaction along the diet–inflammation–stroke axis (56). The combination of impaired vascular function, dyslipidemia, and hypertension resulting from a high-DII diet significantly heightens the risk of stroke, emphasizing the importance of dietary interventions to mitigate this risk by reducing inflammation and promoting vascular health.

### 4.5 Limitations

This study has several limitations. First, the cross-sectional design of the NHANES data limits the ability to establish causality between the DII and stroke risk in MHO individuals. Additionally, self-reported dietary data may introduce biases and inaccuracies, potentially leading to misclassification of dietary exposures. Another limitation is the exclusion of certain food parameters from the DII calculation, which may affect the comprehensiveness of the inflammatory assessment. Specifically, only a subset of 45 possible food parameters was available, and the exclusion of foods with known anti-inflammatory properties may have led to an overestimation of the pro-inflammatory potential of the diets. A further limitation is the relative age homogeneity of our cohort, which restricts generalizability to older adults who bear a higher stroke burden. Lastly, the DII does not account for dietary supplementation or variations in food quantity determination, which could affect the precision of the inflammatory assessment. The large age range within the population, including older adults with heightened inflammatory responses, may also influence the results, contributing to variations in the observed associations.

### 4.6 Clinical implications

DII may serve as a valuable predictive tool in clinical settings, particularly for assessing stroke risk in MHO individuals. By incorporating DII scores into routine assessments, clinicians can identify individuals at higher risk of stroke due to diet-induced inflammation, enabling early interventions. Personalized dietary recommendations based on DII scores could help mitigate stroke risk by promoting anti-inflammatory diets, thus offering a tailored approach to stroke prevention. In our spline analysis, a cut-point of approximately 2.0 DII units emerged: Below this level each 1-unit increase raised stroke odds by 19%, whereas above it the curve flattened. In practical terms, DII ≈ 2.0 corresponded to the upper quartile of our cohort and reflected diets high in processed meat, refined grains. We therefore propose a traffic-light framework—low (< 0), moderate (0 to < 2), and high (≥ 2)—to flag patients who would benefit most from nutrition counseling. Before bedside adoption, however, this threshold needs validation in prospective cohorts and integration with overall cardiovascular-risk assessment.

### 4.7 Future research directions

Future studies should focus on exploring the underlying biological mechanisms linking DII to stroke, particularly the role of inflammation in vascular health. Longitudinal studies are essential to establish the causality of the observed associations between DII and stroke risk. Additionally, intervention trials examining the effects of modifying dietary inflammation through DII-targeted dietary changes could provide valuable insights into the potential for dietary interventions to reduce stroke risk in high-risk populations. Future research should also recruit a broader age spectrumsk. Additionally, inter ≥ 65 years—to validate the age-specific applicability of the observed DII–stroke relationship.

## 5 Conclusion

This study demonstrates a non-linear association between dietary inflammatory potential and stroke risk in metabolically healthy obese adults. The relationship is steepest when DII is below roughly 2.0; beyond that cut-point additional pro-inflammatory load confers little extra risk, suggesting a saturation effect. Incorporating DII—particularly identifying individuals whose scores are at or above this threshold—into routine clinical evaluations may facilitate targeted dietary interventions aimed at reducing stroke risk.

## Data Availability

Publicly available datasets were analyzed in this study. This data can be found at: https://www.cdc.gov/nchs/nhanes/.

## References

[1] GBD 2019 Stroke Collaborators. Global, regional, and national burden of stroke and its risk factors, 1990-2019: A systematic analysis for the Global burden of disease study 2019. *Lancet Neurol.* (2021) 20:795–820. 10.1016/S1474-4422(21)00252-0 34487721 PMC8443449

[2] Chen Z Iona A Parish S Chen Y Guo Y Bragg F Adiposity and risk of ischaemic and haemorrhagic stroke in 0⋅5 million Chinese men and women: A prospective cohort study. *Lancet Glob Health.* (2018) 6:e630–40. 10.1016/S2214-109X(18)30216-X 29773119 PMC5960068

[3] Powell-Wiley T Poirier P Burke L Després J Gordon-Larsen P Lavie C Obesity and cardiovascular disease: A scientific statement from the American heart association. *Circulation.* (2021) 143:e984–1010. 10.1161/CIR.0000000000000973 33882682 PMC8493650

[4] Mauriège P Karelis A Taleb N Clément A Joanisse D. Comparing an adiposopathy approach with four popular classifications schemes to categorize the metabolic profile of postmenopausal women. *J Physiol Biochem.* (2020) 76:609–22. 10.1007/s13105-020-00766-w 32970306

[5] Wang J Xia P Ma M Li Y Geng T Zhang Y Trends in the prevalence of metabolically healthy obesity among US adults, 1999-2018. *JAMA Netw Open.* (2023) 6:e232145. 10.1001/jamanetworkopen.2023.2145 36892842 PMC9999245

[6] Zembic A Eckel N Stefan N Baudry J Schulze M. An empirically derived definition of metabolically healthy obesity based on risk of cardiovascular and total mortality. *JAMA Netw Open.* (2021) 4:e218505. 10.1001/jamanetworkopen.2021.8505 33961036 PMC8105750

[7] Blüher M. Metabolically healthy obesity. *Endocr Rev.* (2020) 41:bnaa004. 10.1210/endrev/bnaa004 32128581 PMC7098708

[8] Perone F Spadafora L Pratesi A Nicolaio G Pala B Franco G Obesity and cardiovascular disease: Risk assessment, physical activity, and management of complications. *Int J Cardiol Cardiovasc Risk Prev.* (2024) 23:200331. 10.1016/j.ijcrp.2024.200331 39346126 PMC11439555

[9] Caleyachetty R Thomas G Toulis K Mohammed N Gokhale K Balachandran K Metabolically healthy obese and incident cardiovascular disease events among 3.5 million men and women. *J Am Coll Cardiol.* (2017) 70:1429–37. 10.1016/j.jacc.2017.07.763 28911506

[10] Wang X Xu Q Liu W Xiong J Li H Xiong N Dietary inflammatory index and its associations with cardiovascular diseases and cancer: Evidence form NHANES 2017-2018 and Mendelian randomization analysis. *Exp Gerontol.* (2025) 199:112665. 10.1016/j.exger.2024.112665 39701432

[11] Zuercher M Harvey D Santiago-Torres M Au L Shivappa N Shadyab A Dietary inflammatory index and cardiovascular disease risk in Hispanic women from the Women’s Health Initiative. *Nutr J.* (2023) 22:5. 10.1186/s12937-023-00838-9 36631866 PMC9835220

[12] Su Z Efremov L Mikolajczyk R. Differences in the levels of inflammatory markers between metabolically healthy obese and other obesity phenotypes in adults: A systematic review and meta-analysis. *Nutr Metab Cardiovasc Dis.* (2024) 34:251–69. 10.1016/j.numecd.2023.09.002 37968171

[13] Abdurahman A Azadbakhat L Rasouli M Chamari M Qorbani M Dorosty A. Association of dietary inflammatory index with metabolic profile in metabolically healthy and unhealthy obese people. *Nutr Diet.* (2019) 76:192–8. 10.1111/1747-0080.12482 30402959

[14] Phillips C Chen L Heude B Bernard J Harvey N Duijts L Dietary inflammatory index and non-communicable disease risk: A narrative review. *Nutrients.* (2019) 11:1873. 10.3390/nu11081873 31408965 PMC6722630

[15] Karelis A Faraj M Bastard J St-Pierre D Brochu M Prud’homme D The metabolically healthy but obese individual presents a favorable inflammation profile. *J Clin Endocrinol Metab.* (2005) 90:4145–50. 10.1210/jc.2005-0482 15855252

[16] Liu Q Bai B Liu F Chen Y Wang Y Wang H Long-term trends in risk factor management in respondents with chronic kidney disease in the USA. *Am J Nephrol.* (2022) 53:614–23. 10.1159/000525733 36126645

[17] Kanagasabai T Thakkar N Kuk J Churilla J Ardern C. Differences in physical activity domains, guideline adherence, and weight history between metabolically healthy and metabolically abnormal obese adults: A cross-sectional study. *Int J Behav Nutr Phys Act.* (2015) 12:64. 10.1186/s12966-015-0227-z 25982079 PMC4490726

[18] Shivappa N Steck S Hurley T Hussey J Hébert J. Designing and developing a literature-derived, population-based dietary inflammatory index. *Public Health Nutr.* (2014) 17:1689–96. 10.1017/S1368980013002115 23941862 PMC3925198

[19] Zhao S Su Y Yang H. Associations of dietary inflammation index and composite dietary antioxidant index with all-cause mortality in COPD patients. *Front Nutr.* (2025) 12:1514430. 10.3389/fnut.2025.1514430 39906240 PMC11790435

[20] Tabung F Steck S Zhang J Ma Y Liese A Agalliu I Construct validation of the dietary inflammatory index among postmenopausal women. *Ann Epidemiol.* (2015) 25:398–405. 10.1016/j.annepidem.2015.03.009 25900255 PMC4433562

[21] Wirth M Shivappa N Davis L Hurley T Ortaglia A Drayton R Construct validation of the dietary inflammatory index among African Americans. *J Nutr Health Aging.* (2017) 21:487–91. 10.1007/s12603-016-0775-1 28448077 PMC5547883

[22] Vahid F Shivappa N Faghfoori Z Khodabakhshi A Zayeri F Hebert J Validation of a dietary inflammatory index (DII) and Association with risk of gastric cancer: A case-control study. *Asian Pac J Cancer Prev.* (2018) 19:1471–7. 10.22034/APJCP.2018.19.6.1471 29936717 PMC6103570

[23] Hajji-Louati M Gelot A Frenoy P Laouali N Guénel P Romana Mancini F. Dietary Inflammatory Index and risk of breast cancer: Evidence from a prospective cohort of 67,879 women followed for 20 years in France. *Eur J Nutr.* (2023) 62:1977–89. 10.1007/s00394-023-03108-w 36869910

[24] Matsumoto Y Shivappa N Sugioka Y Tada M Okano T Mamoto K Change in dietary inflammatory index score is associated with control of long-term rheumatoid arthritis disease activity in a Japanese cohort: The tomorrow study. *Arthritis Res Ther.* (2021) 23:105. 10.1186/s13075-021-02478-y 33832530 PMC8028141

[25] Jung S Papp J Sobel E Pellegrini M Yu H Zhang Z. Pro-inflammatory cytokine polymorphisms and interactions with dietary alcohol and estrogen, risk factors for invasive breast cancer using a post genome-wide analysis for gene-gene and gene-lifestyle interaction. *Sci Rep.* (2021) 11:1058. 10.1038/s41598-020-80197-1 33441805 PMC7807068

[26] Mendon P Witsch M Becker M Adamski A Vaillant M. Facilitating comprehensive child health monitoring within REDCap - An open-source code for real-time Z-score assessments. *BMC Med Res Methodol.* (2024) 24:298. 10.1186/s12874-024-02405-0 39639200 PMC11619695

[27] Muntner P Shimbo D Carey R Charleston J Gaillard T Misra S Measurement of blood pressure in humans: A scientific statement from the American heart association. *Hypertension.* (2019) 73:e35–66. 10.1161/HYP.0000000000000087 30827125 PMC11409525

[28] Kidney Disease: Improving Global Outcomes (KDIGO) CKD Work Group. KDIGO 2024 clinical practice guideline for the evaluation and management of chronic kidney disease. *Kidney Int.* (2024) 105:S117–314. 10.1016/j.kint.2023.10.018 38490803

[29] Zhang Z. Multiple imputation with multivariate imputation by chained equation (MICE) package. *Ann Transl Med.* (2016) 4:30. 10.3978/j.issn.2305-5839.2015.12.63 26889483 PMC4731595

[30] Ji M Hong X Chen M Chen T Wang J Zhang N. Dietary inflammatory index and cardiovascular risk and mortality: A meta-analysis of cohort studies. *Medicine (Baltimore).* (2020) 99:e20303. 10.1097/MD.0000000000020303 32443378 PMC7253850

[31] Mao Y Weng J Xie Q Wu L Xuan Y Zhang J Association between dietary inflammatory index and Stroke in the US population: Evidence from NHANES 1999-2018. *BMC Public Health.* (2024) 24:50. 10.1186/s12889-023-17556-w 38166986 PMC10763382

[32] Yang M Miao S Hu W Yan J. Association between the dietary inflammatory index and all-cause and cardiovascular mortality in patients with atherosclerotic cardiovascular disease. *Nutr Metab Cardiovasc Dis.* (2024) 34:1046–53. 10.1016/j.numecd.2023.11.015 38218715

[33] Ahmad A Lim L Morieri M Tam C Cheng F Chikowore T Precision prognostics for cardiovascular disease in Type 2 diabetes: A systematic review and meta-analysis. *Commun Med (Lond).* (2023) 4:11. 10.1038/s43856-023-00429-z 38253823 PMC10803333

[34] Shivappa N Godos J Hébert J Wirth M Piuri G Speciani A Dietary inflammatory index and cardiovascular risk and mortality-A meta-analysis. *Nutrients.* (2018) 10:200. 10.3390/nu10020200 29439509 PMC5852776

[35] Huang R Lai F Zhao L Zhang J Chen H Wang S Associations between dietary inflammatory index and stroke risk: Based on NHANES 2005-2018. *Sci Rep.* (2024) 14:6704. 10.1038/s41598-024-57267-9 38509177 PMC10954724

[36] Wu B Qiu L Lin Y Lin Q Pan Y. The association between the dietary inflammatory index and cardiorespiratory fitness in United States young adults: A cross-sectional study from the National health and nutrition examination study, 1999-2004. *Front Nutr.* (2024) 11:1442710. 10.3389/fnut.2024.1442710 39391678 PMC11464452

[37] Zhang J Jia J Lai R Wang X Chen X Tian W Association between dietary inflammatory index and atherosclerosis cardiovascular disease in U.S. adults. *Front Nutr.* (2022) 9:1044329. 10.3389/fnut.2022.1044329 36687707 PMC9849765

[38] Liu W Fan L Shi D Yu L Song J Liang R Physiological measures variability and risks of heart disease and stroke: Evidence from three cohort studies. *BMC Med.* (2024) 22:590. 10.1186/s12916-024-03805-1 39695684 PMC11657114

[39] Pinto L Aranha L Luiz R Oliveira GMMD Rosa G. Association of dietary inflammatory potential in metabolically healthy and metabolically unhealthy obese individuals. *Int J Cardiovasc Sci.* (2023) 36:e20230102. 10.36660/ijcs.20230102

[40] Yu X Pu H Voss M. Overview of anti-inflammatory diets and their promising effects on non-communicable diseases. *Br J Nutr.* (2024) 132:898–918. 10.1017/S0007114524001405 39411832 PMC11576095

[41] Delpino F Figueiredo L da Silva B da Silva T Mintem G Bielemann R Omega-3 supplementation and diabetes: A systematic review and meta-analysis. *Crit Rev Food Sci Nutr.* (2022) 62:4435–48. 10.1080/10408398.2021.1875977 33480268

[42] MacDonald C Madika A Gomes R Severi G Sibon I Debette S Physical activity and stroke among women - A non-linear relationship. *Prev Med.* (2021) 150:106485. 10.1016/j.ypmed.2021.106485 33647351

[43] Rasaei N Fatemi S Gholami F Samadi M Mohammadian M Daneshzad E Interaction of genetics risk score and fatty acids quality indices on healthy and unhealthy obesity phenotype. *BMC Med Genomics.* (2025) 18:16. 10.1186/s12920-024-02066-4 39838481 PMC11753101

[44] Sharma I Zhu Y Woodrow J Mulay S Parfrey P Mclaughlin J Inflammatory diet and risk for colorectal cancer: A population-based case-control study in Newfoundland. *Canada. Nutrition.* (2017) 42:69–74. 10.1016/j.nut.2017.05.010 28870481

[45] Chang Y Yu C Dai X Sun H Tang T. Association of dietary inflammatory index and dietary oxidative balance score with gastrointestinal cancers in NHANES 2005-2018. *BMC Public Health.* (2024) 24:2760. 10.1186/s12889-024-20268-4 39385181 PMC11465896

[46] Chee Y Dalan R Cheung C. The interplay between immunity, inflammation and endothelial dysfunction. *Int J Mol Sci.* (2025) 26:1708. 10.3390/ijms26041708 40004172 PMC11855323

[47] Dakal T Xiao F Bhusal C Sabapathy P Segal R Chen J Lipids dysregulation in diseases: Core concepts, targets and treatment strategies. *Lipids Health Dis.* (2025) 24:61. 10.1186/s12944-024-02425-1 39984909 PMC11843775

[48] Heidarzadeh-Esfahani N Hajahmadi S Pasdar Y Darbandi M Najafi F Moradinazar M Diet-related inflammation is positively associated with atherogenic indices. *Sci Rep.* (2024) 14:13190. 10.1038/s41598-024-63153-1 38851843 PMC11162500

[49] Bhol N Bhanjadeo M Singh A Dash U Ojha R Majhi S The interplay between cytokines, inflammation, and antioxidants: Mechanistic insights and therapeutic potentials of various antioxidants and anti-cytokine compounds. *Biomed Pharmacother.* (2024) 178:117177. 10.1016/j.biopha.2024.117177 39053423

[50] Wang A Wang F Huang Y Cui Q Xu Y Zhang W Association between systemic inflammatory markers and all-cause mortality in patients with stroke: A prospective study using data from the UK Biobank. *J Stroke Cerebrovasc Dis.* (2024) 33:108076. 10.1016/j.jstrokecerebrovasdis.2024.108076 39393512

[51] Caratis F Karaszewski B Klejbor I Furihata T Rutkowska A. Differential expression and modulation of EBI2 and 7α,25-OHC synthesizing (CH25H, CYP7B1) and degrading (HSD3B7) enzymes in mouse and human brain vascular cells. *PLoS One.* (2025) 20:e0318822. 10.1371/journal.pone.0318822 39999050 PMC11856462

[52] Kleeberg A Luft T Golkowski D Purrucker J. Endothelial dysfunction in acute ischemic stroke: A review. *J Neurol.* (2025) 272:143. 10.1007/s00415-025-12888-6 39812851 PMC11735568

[53] Thilarajah S Mentiplay B Bower K Tan D Pua Y Williams G Factors associated with post-stroke physical activity: A systematic review and meta-analysis. *Arch Phys Med Rehabil.* (2018) 99:1876–89. 10.1016/j.apmr.2017.09.117 29056502

[54] Sahabudhee A Rao C Chandrasekaran B Pedersen S. Dose-response effects of periodic physical activity breaks on the chronic inflammatory risk associated with sedentary behavior in high- and upper-middle income countries: A systematic review and meta-analysis. *Diabetes Metab Syndr.* (2023) 17:102730. 10.1016/j.dsx.2023.102730 36863092

[55] Bull F Al-Ansari S Biddle S Borodulin K Buman M Cardon G World health organization 2020 guidelines on physical activity and sedentary behaviour. *Br J Sports Med.* (2020) 54:1451–62. 10.1136/bjsports-2020-102955 33239350 PMC7719906

[56] Khera A Chaffin M Aragam K Haas M Roselli C Choi S Genome-wide polygenic scores for common diseases identify individuals with risk equivalent to monogenic mutations. *Nat Genet.* (2018) 50:1219–24. 10.1038/s41588-018-0183-z 30104762 PMC6128408

